# The role of tetramethylpyrazine and paeoniflorin in modulating iron metabolism and ferroptosis: innovative strategies for atherosclerosis treatment

**DOI:** 10.3389/fphar.2026.1845893

**Published:** 2026-07-13

**Authors:** Miao Zhang, WenBo Wei, ZongZheng Chen, ShiQiang Yan, ZhengZhi Wu, ZhiXiu Lin, HuiJun Yin, FengQin Xu

**Affiliations:** 1 School of Chinese Medicine, Faculty of Medicine, The Chinese University of Hong Kong, Hong Kong, Hong Kong SAR, China; 2 Shenzhen Second People’s Hospital, First Affiliated Hospital of Shenzhen University, Shenzhen, Guangdong, China; 3 Center of Cancer Immunology, Shenzhen Institute of Advanced Technology Chinese Academy of Sciences, Shenzhen, Guangdong, China; 4 Department of Cardiovascular Disease, Beijing Xiyuan Hospital, China Academy of Chinese Medical Sciences, Beijing, China

**Keywords:** atherosclerosis, ferroptosis, iron overload, paeoniflorin, tetramethylpyrazine

## Abstract

**Introduction:**

Atherosclerosis (AS) is driven by lipid accumulation, inflammation, and oxidative stress, leading to endothelial dysfunction and plaque formation. Emerging evidence indicates that iron overload and ferroptosis exacerbate these processes via enhanced lipid peroxidation and vascular injury. However, current Western medical strategies predominantly target lipid-lowering and inflammation, often overlooking iron dysregulation and ferroptotic pathways.

**Methods:**

We used ApoE^−/−^ mice as an AS model and administered Tetramethylpyrazine (TMP), Paeoniflorin (PF), or their combination (TMP+PF). Serum lipids, oxidative stress biomarkers, iron metabolism indices, and ferroptosis-related markers were measured.

**Results:**

Both TMP and PF significantly reduced serum lipid levels and oxidative stress, normalized iron metabolism parameters, and suppressed ferroptosis-associated markers, indicating a protective effect against ferroptotic damage.

**Discussion:**

These findings suggest that TMP and PF mitigate AS by coordinately regulating oxidative stress, iron homeostasis, and ferroptosis. Their multi-targeted action may offer a complementary approach to conventional therapies, addressing the previously neglected mechanisms of iron-driven vascular pathology.

## 1 Introduction

Atherosclerosis (AS) is a chronic inflammatory disease characterized by lipid, inflammatory cell, and fibrous element accumulation in the arterial wall, leading to plaque formation (Baumer et al., 2025). It significantly contributes to cardiovascular diseases, including heart attacks and strokes (Libby, 2021). The progression of AS involves complex interactions among risk factors such as hyperlipidemia, hypertension, diabetes, and lifestyle choices like smoking and poor diet (Xu et al., 2025). Despite advances in understanding AS, effective treatments remain limited (Duntas and Feldt-Rasmussen, 2026). Traditional lipid-lowering therapies focus on LDL cholesterol but neglect other factors like oxidative stress, iron metabolism, and ferroptosis (Schade et al., 2026). Recent research highlights the importance of these factors in the pathogenesis of AS, suggesting new avenues for intervention.

Reactive oxygen species (ROS) are highly reactive molecules that damage cellular metabolites, contributing to oxidative stress in AS (Kattoor et al., 2017). Elevated ROS levels promote endothelial dysfunction, inflammation, and smooth muscle cell proliferation, while also oxidizing LDL particles into more atherogenic oxidized LDL (oxLDL), which enhances foam cell formation and plaque development (Förstermann et al., 2017). Excess iron, though essential, catalyzes ROS production and exacerbates oxidative damage and inflammation in the arterial wall, further linked to increased lipid peroxidation and foam cell formation (Guo et al., 2023). Abnormal levels of hepcidin (HEP) and ferroportin (FPN) disrupt iron homeostasis, leading to iron overload, which amplifies ROS production and worsens inflammation, ultimately promoting plaque instability (Jayakumar et al., 2022). Additionally, ferroptosis—a regulated form of cell death driven by lipid peroxidation and iron dependency—can lead to the death of vascular cells, aggravating plaque instability (Zheng et al., 2022). The interplay between oxidative stress, iron overload, and ferroptosis creates a vicious cycle that promotes further damage and inflammation, ultimately driving the progression of AS (Xu X. et al., 2024; Jinson et al., 2025).

Traditional Chinese Medicine (TCM) has been increasingly recognized for its potential in managing cardiovascular diseases, including AS (Zhi et al., 2023). Among the various botanical metabolites used in TCM, Tetramethylpyrazine (TMP) and Paeoniflorin (PF) have garnered attention for their therapeutic properties (Yuan et al., 2018). TMP, derived from the Chinese botanical drug Ligusticum chuanxiong, has antioxidant and anti-inflammatory effects. It can improve endothelial function, reduce oxidative stress, and inhibit platelet aggregation, which are crucial in preventing the progression of AS (Li et al., 2022). Research has shown that TMP can lower cholesterol levels, improve blood flow, and protect vascular cells from oxidative damage (Zhang et al., 2022). By enhancing the body’s antioxidant defenses, TMP helps mitigate the harmful effects of ROS and iron overload, both of which are implicated in AS (Zhang et al., 2019). Clinical studies have demonstrated that TMP can improve cardiac function and reduce symptoms in patients with cardiovascular diseases, supporting its role as a complementary therapy in managing AS (Chen et al., 2020). PF, a key active metabolite of the peony root (*Paeonia lactiflora*), possesses anti-inflammatory, antioxidant, and cardioprotective properties (Ren et al., 2023). It helps regulate lipid metabolism and has been shown to reduce lipid accumulation in vascular tissues (Zhou et al., 2020). PF not only lowers cholesterol levels but also protects endothelial cells from injury and apoptosis. Its ability to inhibit inflammatory pathways contributes to the stabilization of atherosclerotic plaques, reducing the risk of cardiovascular events (Yu et al., 2022). Studies indicate that PF can improve endothelial function and reduce markers of inflammation in patients with AS, highlighting its potential as a therapeutic agent in cardiovascular health (Zhang and Wei, 2020).

TMP and PF have shown potential in improving cardiovascular disease(CVD) by targeting iron overload and ferroptosis. These metabolites help restore iron homeostasis by reducing excess iron levels, which may decrease the production of ROS that contribute to oxidative stress and inflammation in CVD (Wu et al., 2024; Sun et al., 2018), these results suggest that TMP and PF have promising therapeutic potential against AS. TMP and PF exert protective effects on vascular cells, potentially preventing ferroptosis and preserving endothelial function, thereby enhancing vascular stability and reducing plaque vulnerability (Wang et al., 2017; Xu M. et al., 2024). Additionally, both TMP and PF possess antioxidant and anti-inflammatory properties, which further stabilize plaques and improve overall cardiovascular health by mitigating oxidative stress and inflammation (Yuan et al., 2018). Previous research highlights the potential of TMP and PF in improving AS through their antioxidant, anti-inflammatory, and lipid-regulating properties, as well as their roles in modulating iron overload and ferroptosis (Zhang et al., 2019; Wu et al., 2024; Chen et al., 2025). These findings support the continued exploration of these metabolites as therapeutic options in cardiovascular disease management. Further clinical studies are needed to establish their efficacy and safety in human populations.

TMP and PF have shown promise in treating AS by targeting various pathophysiological processes. They decrease lipid accumulation in arterial walls, enhance endothelial function through increased nitric oxide availability, and exhibit anti-inflammatory and antioxidant properties (Wang et al., 2017). This study aims to explore the effects of TMP and PF on AS, particularly their roles in inhibiting ROS, iron accumulation, and ferroptosis. We hypothesize that these metabolites will significantly reduce plaque formation and improve vascular health by mitigating oxidative stress and regulating iron homeostasis. To test this, we will use an ApoE^-/-^ mouse model on a high-fat diet to induce AS. This will enable us to examine the effects of TMP and PF on atherosclerotic lesions and lipid profiles *in vivo*, closely resembling human AS (Emini et al., 2017). The study intends to provide insights into the therapeutic potential of TMP and PF for managing AS. And it is the first study to systematically investigate the effects of TMP and PF on ferroptosis-associated markers and iron metabolism-related parameters in AS, as well as their potential protective effects, which have not been previously reported.

## 2 Materials and methods

### 2.1 Chemicals, drugs and reagents

**FIGURE 1:**
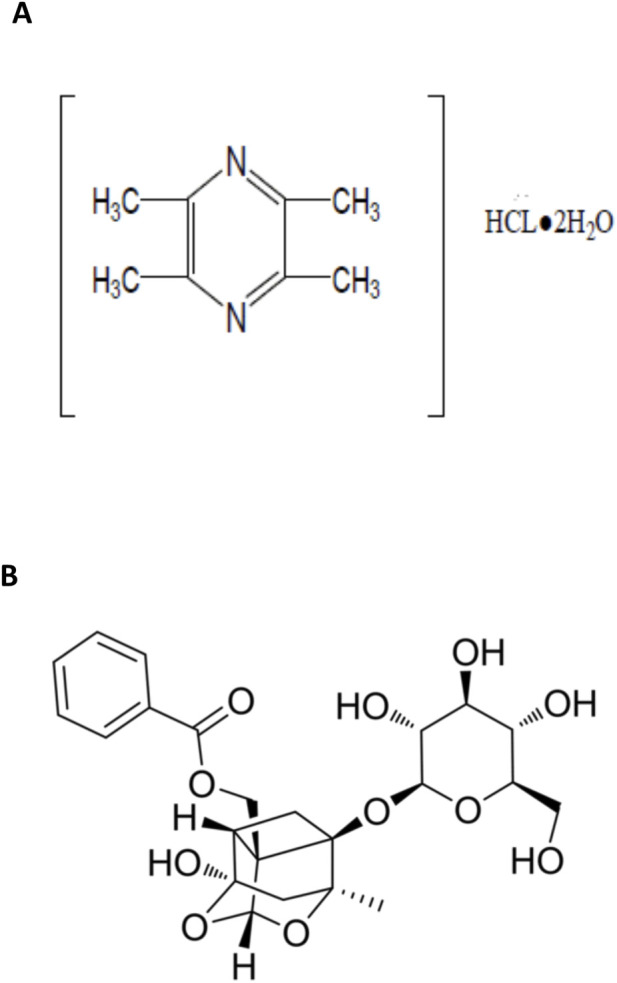
The chemical formula of Tetramethylpyrazine [TMP **(A)**] and Paeoniflorin [PF, **(B)**] used in the current study.

Pure natural metabolites extracted from Chinese medicinal plants were sourced from the National Institutes for Food and Drug Control in Beijing, China, with a purity exceeding 99.5%. The metabolites include TMP (C_8_H_12_N_2_·HCl·2H_2_O, Figure 1) and PF (C_23_H_28_O11, Figure 1). Dimethyl sulfoxide (DMSO) from Solarbio (cat.no.120-51-4, Beijing, China) was utilized to dissolve these metabolites, maintaining a final DMSO concentration of less than 0.5%. Simvastatin (MK-733, cat.no.79902-63-9) was purchased from Jingxin Pharmaceutical Industry, located in Zhejiang, China. The serum Hepcidin(HEP, cat.no.AE92047Mu), ferroportin (FPN, cat.no.RD-FPN-Hu) reagents were purchased from AMEKO (Lianshuo Biotechnology Co., Ltd.,Shanghai, China). Total cholesterol (TC, cat.no. A111-2-1) and triglyceride (TG, cat.no. A110-1-1), ROS(cat.no. E004-1-1), malondialdehyde (MDA, cat.no. A003-1–2), catalase (CAT, cat.no. A007-1-1) and superoxide dismutase (SOD, cat.no. A001-3-2) ELISA kits and tissue iron kit were purchased from Nanjing Jiancheng Technology Co., Ltd.

### 2.2 Animal modelling, grouping, treatment and tissue harvesting

Animal experiments were approved by Chongqing Western Biomedical Technology Co., Ltd. Animal Care and Use Ethics Committee (Approval No. WST.No20211230C0441231[1]) following ARRIVE 2.0 guidelines. Homozygous C57BL/6 and ApoE gene knockout (ApoE^-/-^) mice were purchased from the Chongqing Western Biomedical Technology Co., Ltd. (Chongqing, China), and housed in a central specific pathogen-free (SPF) facility. 36 ApoE gene knockout (ApoE^-/-^) mice (18 males + 18 females, 4–5 weeks old, 17–20g, 7 to 8 per group) were adapted to regular feed for 1 week, then divided into five groups for high-fat feeding over 4 weeks to establish an AS model. Eight SPF-grade C57BL/6 mice (4 males + 4 females, 4–5 weeks old, 18–20g) were fed regular feed for 4 weeks. The mice used a computer-generated random number sequence divided into 6 groups: control, AS model, MK-733, TMP, PF and TMP + PF treatment group. The control group consisted of C57 mice on normal diet and daily gavage with saline (0.9% NaCl). The model and treatment groups used ApoE^-^/^-^ mice, which were fed high-fat diet (60% kcal fat) and continued this diet throughout the 12-week treatment period, receiving daily drug gavage. The dose of MK-733 was 2.5 mg/kg/day, while the dosage for the other three natural metabolite groups was 25 mg/kg/day, the selected dose was based on previous literature (Zhang et al., 2017; Yu et al., 2022; Yuan et al., 2024), administered for 12 weeks. TMP and PF were first dissolved in a minimal amount of DMSO and then diluted with saline to a final DMSO concentration of 0.1% (TMP and PF was dissolved in 10 μL of DMSO and diluted with saline to a final volume of 10 mL, giving a final DMSO concentration of 0.1%), whereas MK-733 was diluted directly with water. MK-733, more commonly known as simvastatin, is a well-characterized oral HMG-CoA reductase inhibitor that has been clinically approved for lipid-lowering management and cardiovascular disease prevention for decades. Mice were anesthetized with pentobarbital sodium (50 mg/kg, i.p.) 24 h after the last drug dose, then euthanized by exsanguination via cardiac puncture, and the entire aorta (∼18 mm) was isolated for Oil Red O staining. For HE staining, the aortic root was additionally collected, fixed, and processed for paraffin embedding. Blinding was not used during treatment, sample collection, histological evaluation, or data analysis.

### 2.3 Blood biochemical index testing

Mouse serum was analyzed using a full-automatic biochemical analyzer to measure lipid indices, including triglycerides (TG), total cholesterol (TC), High-Density Lipoprotein Cholesterol (HDL-C), Low-Density Lipoprotein Cholesterol (LDL-C), small dense low-density lipoprotein (SD-LDL), C-reactive protein (CRP) and interleukin-6 (IL-6) following reagent manual instructions. Serum levels of Fe, HEP, FPN, ROS, MDA, CAT and SOD were measured using the ELISA method, adhering to Nanjing Jiancheng kit instructions.

### 2.4 Histopathological examination

Aortic and liver tissues were fixed for 7 days and then paraffin-embedded to prepare sections for hematoxylin and eosin (HE) staining, while Oil Red O staining was performed on fresh-frozen sections specifically to observe lipid deposition.

### 2.5 Detection of iron content in tissue

Mouse aorta and liver tissue was homogenized in physiological saline (10%), centrifuged at 3,000 rpm for 10 min, and the supernatant (500 μL) was collected for iron content testing, following kit instructions.

### 2.6 Western blotting

Antibodies for HEP(cat.no.29572), FPN(cat.no.80672), GPX4(cat.no.52455), P53(cat.no.9282), SLC7A11(cat.no.98051), PTGS2(cat.no.12282), FTH1(cat.no.3998) were purchased from Cell Signaling Technology (USA), NOX1(cat.no.ab131088) from Abcam (UK), β-actin(cat.no.sc47778) from Santa Cruz (USA). Liver tissue were lysed in ice-cold lysis buffer (cat.no.BES20728SJ, Biosen, China) with protease inhibitors, and protein concentrations were measured using a BCA protein assay kit (cat.no.23227, Pierce, USA). Total protein (20 μg) was separated by SDS-PAGE and transferred to a PVDF membrane (cat.no.1620177, Biorad, USA). After blocking with 5% nonfat milk in 1× TBST for 1 h at room temperature, membranes were incubated overnight at 4 °C with primary antibodies. Following washes with TBST, they were incubated with a secondary antibody for 1 h at room temperature. Signals were detected using Bio-Rad Clarity™ ECL substrate and captured with the iBright 1500 imaging system (Invitrogen, USA). Data analysis was performed using ImageJ 1.41 software (Bethesda, USA). Band intensities were quantified using ImageJ software. The optical density of each target protein band was first normalized to that of β-actin (loading control) in the same lane, yielding a relative expression value. The normalized values were then expressed as fold-change relative to the control group, which was set to 1.0.

### 2.7 RNA isolation and qRT-PCR

Total RNA was isolated using the EASYspin reagent kit (Biomed Biotech, China, cat.no. RA105-01) following the manufacturer’s protocol. RNA quality and quantity were assessed with a NanoDrop™ One spectrophotometer (Thermo Scientific, USA). First-strand cDNA was synthesized from 1 μg of total RNA using iScript Reverse Transcription Supermix (Vazyme Biotech, China). qRT-PCR analysis was performed with Faststart Essential DNA Green Master (Roche, Switzerland) on a LightCycler 96 Real-Time PCR system (Roche). Primers were designed using Primer3Plus software (Cambridge, USA), with GAPDH as the reference gene. Relative mRNA expression was calculated based on established methods (Zhang et al., 2016). Primer sequences are listed in Supplementary Table 1.

### 2.8 Statistical analysis

Statistical comparisons among the groups were conducted using one-way ANOVA followed by Tukey’s post-hoc test for multiple comparisons, using GraphPad Prism 8.0 software (San Diego,CA, USA). A p-value of less than 0.05 was regarded as statistically significant. Results are expressed as mean ± standard error of the mean (SEM).

## 3 Results

### 3.1 Natural metabolites reduce blood lipid levels and inflammatory factors in AS mice

**FIGURE 2:**
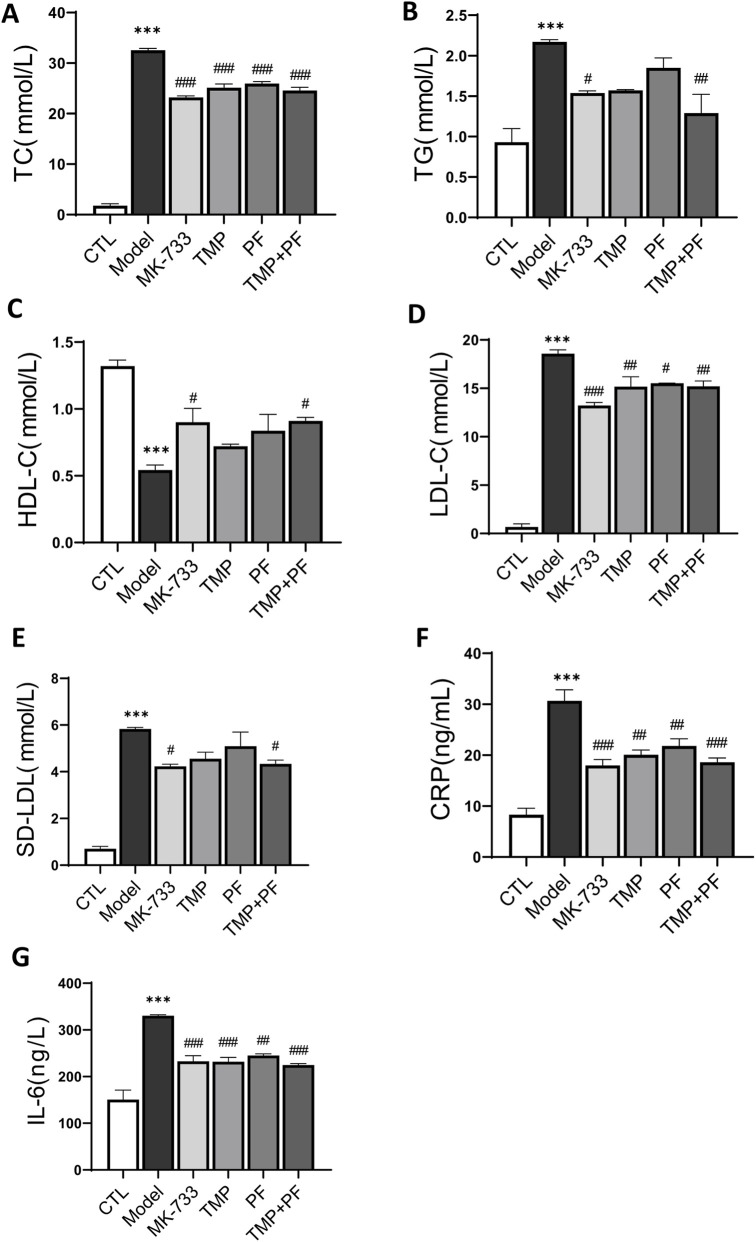
Effects of TMP and PF on lipid levels and inflammation in AS mice. Compared to control, the model group showed significantly elevated TC **(A)** LDL-C **(D)** and the 4 treatment groups had markedly reduced. TG **(B)** HDL-C **(C)** and SD-LDL **(E)** levels were only significantly reversed in the MK-733 and TMP + PF groups. Inflammatory markers CRP **(F)** and IL-6 **(G)** were increased in the model group but substantially decreased in all 4 treatment groups. Data are shown as means ± SEM. Compared with control group, *P < 0. 05, **P < 0. 01, ***P < 0. 001; compared with model group, #*P* < 0. 05, ##*P* < 0. 01, ###*P* < 0. 001. n = 7.

In the model group, TC, LDL-C, and SD-LDL levels were significantly elevated compared to the control group (P <0.05). In contrast, the 4 treatment groups showed significant decreases in TC, LDL-C levels (P <0.05). Additionally, TG and SD-LDL levels only demonstrated significant reductions in the MK-733 and TMP + PF groups, HDL-C also significantly increased only in the MK-733 and TMP + PF groups (P <0.05). Inflammatory markers, such as CRP and IL-6, were elevated in the model group but significantly decreased in the 4 treatment groups (P <0.05), as shown in Figure 2. These findings indicate that these natural metabolites effectively reduced lipid abnormalities and inflammation, highlighting their potential as therapeutic options for managing AS.

### 3.2 Natural metabolites decrease serum iron, HEP, FPN, and tissue iron levels

**FIGURE 3:**
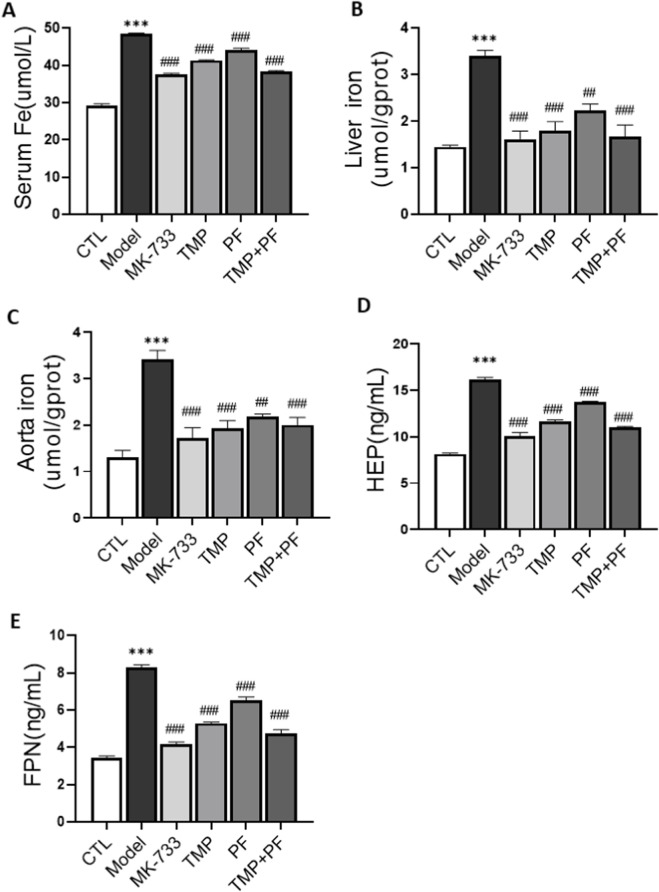
Effects of treatment on iron metabolism in AS mice. The model group exhibited significant increases in serum iron **(A)** liver iron **(B)** aortic iron **(C)** HEP **(D)** FPN **(E)** expression compared to the control group. The 4 treatment groups showed significant reductions compared to the model group. Data are shown as means ± SEM. Compared with control group, *P < 0. 05, **P < 0. 01, ***P < 0. 001; compared with model group, #*P* < 0. 05, ##*P* < 0. 01, ###*P* < 0. 001. n = 7.

We found that, compared to the control group, the model group exhibited significant increases in serum iron, liver iron, aortic iron, HEP and FPN expression (P < 0.01). In contrast, the expression levels of serum iron, liver iron, aortic iron, HEP and FPN in the 4 treatment groups were significantly reduced compared to the model group (P < 0.05), as shown in Figure 3. The serum iron, liver iron, aortic iron, HEP and FPN in the model group indicate disrupted iron homeostasis linked to inflammation and AS. In contrast, the treatment groups showed notable reductions in these markers, suggesting that the natural metabolites effectively restored iron metabolism and reduced iron overload.

### 3.3 Natural metabolites improve oxidative stress response in AS mice

**FIGURE 4:**
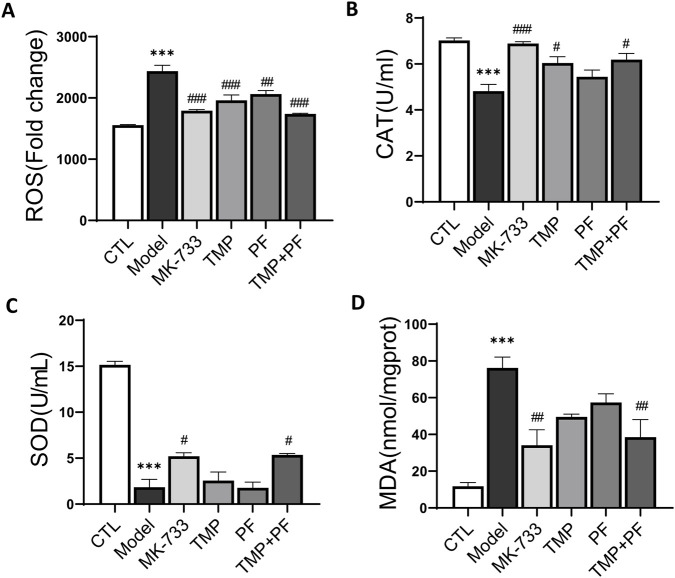
Effects of treatment on oxidative stress markers in AS mice. Compared to the control group, the model group exhibited significant increases in serum ROS **(A)** and MDA **(D)** levels, along with notable decreases in CAT **(B)** and SOD **(C)** levels. In contrast, the 4 treatment groups effectively reversed these changes, with particularly pronounced effects observed in the MK-733 and TMP + PF groups. Data are shown as means ± SEM. Compared with control group, *P < 0. 05, **P < 0. 01, ***P < 0. 001; compared with model group, #*P* < 0. 05, ##*P* < 0. 01, ###*P* < 0. 001. n = 7.

Oxidative stress was assessed by measuring various biomarkers in the mice. Compared to the control group, the model group showed significant increases in serum ROS and MDA levels (P < 0.01), along with notable decreases in CAT and SOD levels (P < 0.01). In contrast, the 4 treatment groups effectively reversed these changes, with the effects being particularly pronounced in the MK-733 and TMP + PF groups (P < 0.05), as shown in Figure 4. This suggests that these treatments enhance the body’s antioxidant defenses, mitigating oxidative damage and improving overall oxidative stress responses in AS.

### 3.4 Natural metabolites improve iron overload and ferroptosis in AS

**FIGURE 5:**
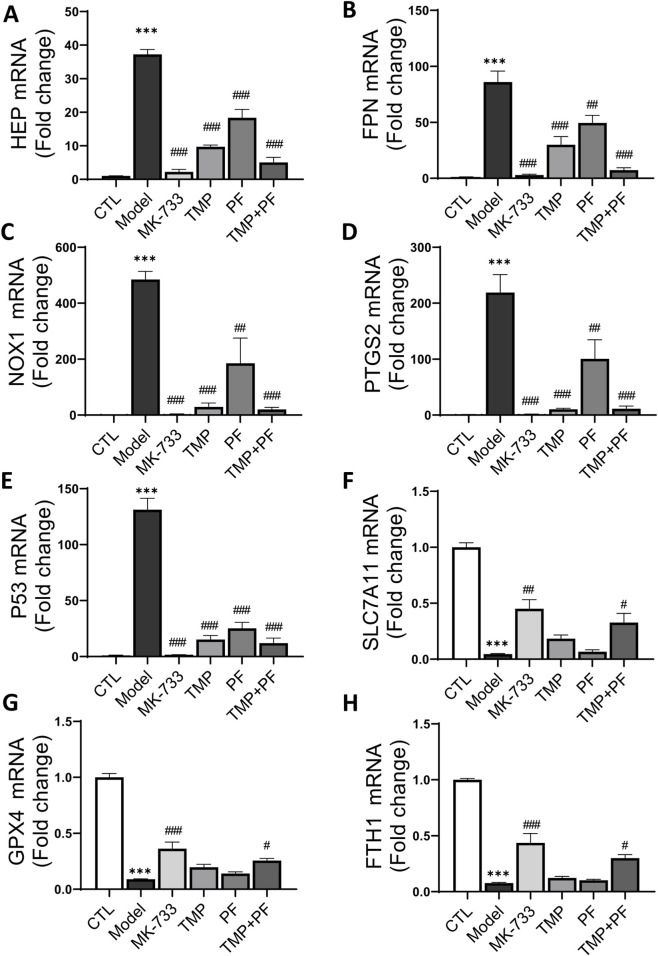
Effects of treatment on iron metabolism and ferroptosis mRNA expression in AS mice. Compared to the control group, the model group showed significant increases in mRNA levels of HEP **(A)** FPN **(B)** NOX1 **(C)** PTGS2 **(D)** and P53 **(E)**. In contrast, SLC7A11 **(F)** GPX4 **(G)** and FTH1 **(H)** mRNA levels were notably decreased. All four treatment groups significantly reversed the abnormal mRNA expression observed in the model group. Data are shown as means ± SEM. Compared with control group, *P < 0. 05, **P < 0. 01, ***P < 0. 001; compared with model group, #*P* < 0. 05, ##*P* < 0. 01, ###*P* < 0. 001. n = 7.

**FIGURE 6:**
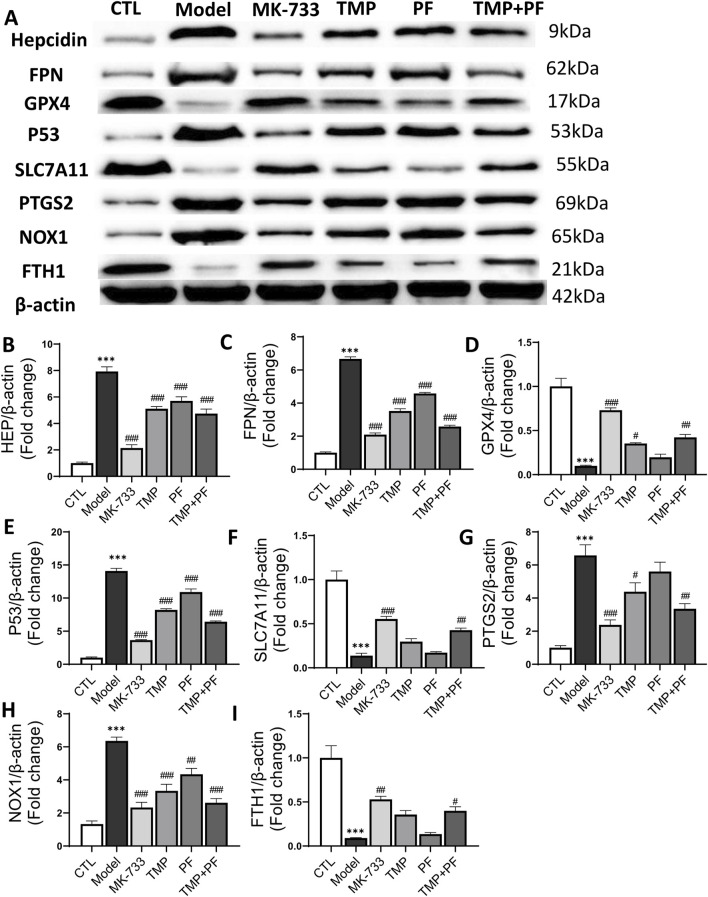
Effects of treatment on iron metabolism and ferroptosis related protein expression levels in AS mice. The model group exhibited significant increases in HEP, FPN, P53, PTGS2, and NOX1 protein levels, while GPX4, SLC7A11, and FTH1 levels were notably decreased compared to the control group. All 4 treatment groups significantly reversed these abnormal protein expressions **(A)**. Quantitative results are shown in **(B–I)**. Data are shown as means ± SEM. Compared with control group, *P < 0. 05, **P < 0. 01, ***P < 0. 001; compared with model group, #*P* < 0. 05, ##*P* < 0. 01, ###*P* < 0. 001. n = 3.

Compared to the control group, the model group exhibited significant increases in the mRNA expression levels of HEP, FPN, NOX1, PTGS2, and P53. Conversely, the expression levels of SLC7A11, GPX4, and FTH1 mRNA were notably decreased. In comparison to the model group, all four treatment groups significantly reversed the abnormal mRNA expression observed in the model group (P < 0.01), as shown in Figure 5. Compared to the control group, the model group exhibited significant increases in the protein expression of HEP, FPN, NOX1, PTGS2, and P53, while the protein levels of SLC7A11, GPX4, and FTH1 were notably decreased. In contrast, all four treatment groups significantly reversed these abnormal protein expressions, consistent with the mRNA results, as shown in Figure 6.

The increased expression of HEP, FPN, P53, PTGS2, and NOX1 in the model group indicates dysregulated iron metabolism and potential ferroptosis, while the decreases in GPX4, SLC7A11, and FTH1 suggest impaired antioxidant defenses. Ferroptosis, driven by lipid peroxidation and iron overload, contributes to oxidative damage in AS. The significant reversal of ferroptosis-associated markers in the treatment groups suggests their potential to modulate iron homeostasis-related parameters, indicating a possible therapeutic value of these natural metabolites in conditions involving ferroptosis-related pathways in AS.

### 3.5 Histological analysis of liver and aortic tissue

**FIGURE 7:**
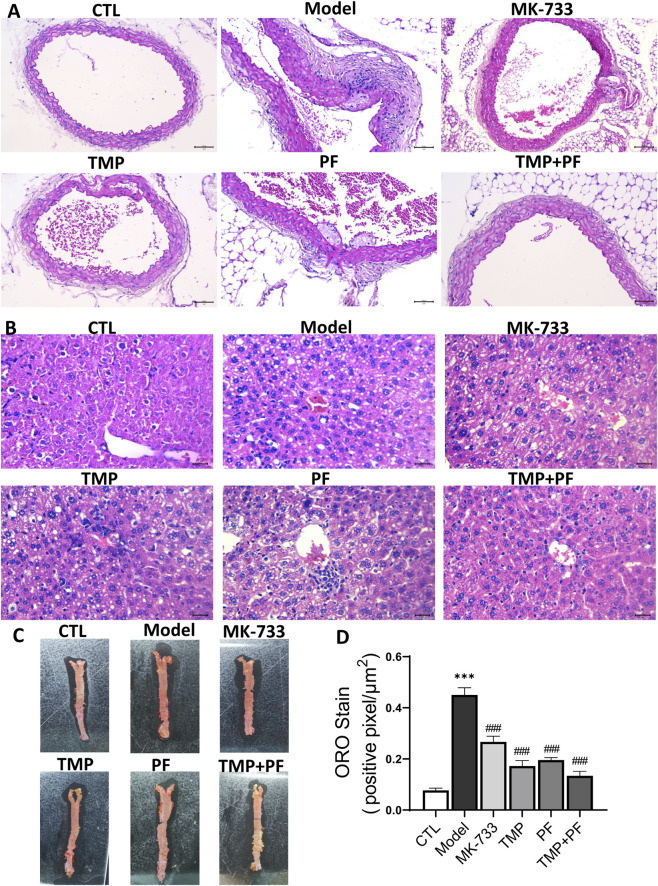
Histological and staining results in AS mice. HE staining of the aorta **(A)** HE staining of the liver **(B)** and Oil Red O staining of the aorta **(C)**. **(D)** Quantitative analysis of lipid deposition (percentage of Oil Red O-positive area). Data are shown as means ± SEM. Compared with control group, *P < 0. 05, **P < 0. 01, ***P < 0. 001; compared with model group, #*P* < 0. 05, ##*P* < 0. 01, ###*P* < 0. 001. n = 5.

HE staining revealed that the control group had clear liver lobule and hepatic cord structures, with orderly cell arrangement and large, round nuclei. In contrast, the model group exhibited unclear lobule structure with diffuse lipid vacuoles, hydropic degeneration, inflammatory cell infiltration, and apoptotic hepatocytes. Drug intervention significantly improved liver tissue, particularly in the positive control and TMP + PF groups (Figure 7B). Similarly, the aorta of the control group showed a smooth intima with intact endothelial cells, while the model and treatment groups exhibited endothelial disruption and media thickening. Post-treatment, the aortic endothelial structure improved markedly, especially in the positive control and TMP + PF groups (Figure 7A). Oil Red O staining results showed an increase in red lipid droplets in the model group, while all four treatment groups exhibited a significant reduction, with the positive control and TMP + PF groups showing the most pronounced decreases (Figures 7C,D). The results offer a comprehensive assessment of tissue health in the model and treatment groups. HE staining revealed structural abnormalities, including disrupted liver lobules and endothelial damage in the model group, while Oil Red O staining indicated increased lipid accumulation, reflecting metabolic dysregulation. Significant improvements post-treatment, with clearer tissue structures and reduced lipid droplets, demonstrate the efficacy of the natural metabolites in restoring tissue health and mitigating lipid overload. This underscores their therapeutic potential in addressing structural and metabolic abnormalities associated with AS.

## 4 Discussion

**FIGURE 8:**
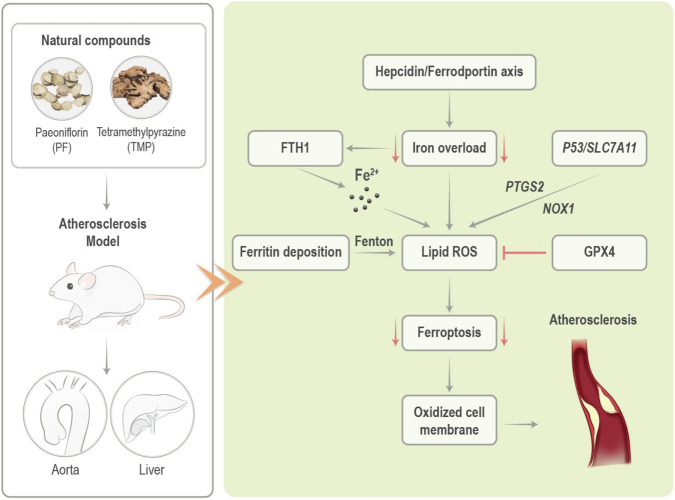
The Protective Effects of TMP and PF on AS and the Underlying Mechanisms. TMP and PF showed potential protective effects against AS, which may be linked to the modulation of inflammatory responses, oxidative stress, and ferroptosis-associated markers, including parameters related to iron metabolism.

The present study revealed that TMP and PF effectively mitigate AS by regulating iron metabolism, reducing blood lipids, and modulating inflammation and oxidative stress. The treatment significantly lowered serum iron levels, HEP, and FPN expression, thereby restoring iron homeostasis disrupted in AS models. Additionally, TMP and PF decreased pro-inflammatory markers and oxidative stress indicators, while improving the expression of genes and proteins related to iron metabolism and ferroptosis. These findings suggest that the protective effects of TMP and PF against AS are mediated by their ability to correct iron dysregulation, lower blood lipid levels, and modulation of ferroptosis-related markers, highlighting their potential as therapeutic agents in managing AS (Figure 8). Notably, in our AS animal models, compensatory transcriptional upregulation of FPN occurs under ferroptotic stress. Although serum hepcidin levels increase, FPN degradation is partially blunted, ultimately leading to elevated total FPN protein levels and resulting in discordance between hepcidin levels and FPN function; the coexistence of elevated hepcidin and increased FPN expression in our AS model suggests context-specific regulation, which may result from increased FPN synthesis, local hepcidin resistance or impaired FPN degradation, and further investigation into FPN localization and iron export activity is required.

Our findings align with previous publications that emphasize the critical link between iron metabolism, ferroptosis, and the pathogenesis of AS (Förstermann et al., 2017; Xu M. et al., 2024; Vinchi et al., 2020). Numerous studies have shown that dysregulated iron homeostasis contributes to increased oxidative stress and inflammation, both of which are fundamental drivers of AS (Kattoor et al., 2017). For instance, elevated serum ferritin and transferrin levels have been associated with a higher risk of cardiovascular diseases, including AS (Silvestre et al., 2017). Additionally, research indicates that iron overload exacerbates lipid peroxidation, leading to endothelial dysfunction and promoting atherosclerotic plaque formation (Guo et al., 2023). Ferroptosis has emerged as a significant factor in the progression of AS (Ouyang et al., 2021). Studies have reported that ferroptosis can lead to the death of endothelial cells and macrophages within atherosclerotic plaques, further destabilizing these lesions (Liu et al., 2022; Ma et al., 2022). Our study adds to this body of literature by suggesting that TMP and PF are associated with modulation of iron homeostasis-related parameters and reduced ferroptosis-associated markers, which may have implications for AS pathophysiology. This contrasts with some studies that primarily focus on the detrimental effects of iron accumulation and ferroptosis without exploring potential therapeutic interventions, thereby providing a more comprehensive understanding of how modulation of iron metabolism and ferroptosis can influence AS progression.

Numerous studies have investigated the cardioprotective effects of TMP and PF in cardiovascular diseases (Pang et al., 2024). TMP has been shown to attenuate myocardial ischemic injury by reducing oxidative stress, improving endothelial function, and promoting angiogenesis, likely through modulation of inflammatory and apoptotic signaling pathways (Li et al., 2022). Additionally, TMP inhibits iron overload, suggesting it may prevent ferroptotic cell death in cardiovascular contexts (Zhang et al., 2019). Similarly, PF has demonstrated protective effects against cardiac injury, enhancing myocardial recovery following ischemic events. It has also been shown to improve lipid profiles and reduce inflammation, indicating its potential to mitigate AS-related complications (Li et al., 2023). Notably, PF may regulate ferroptosis-associated pathways, further contributing to its cardioprotective effects (Wu et al., 2024). Overall, these findings highlight that TMP and PF address iron dysregulation, inflammation, and ferroptosis, positioning them as promising therapeutic candidates for AS and related conditions.

As evidenced by numerous studies, the mechanisms of AS involve interactions between iron overload and ferroptosis signaling pathways (Fang et al., 2023). Iron overload heightens oxidative stress, contributing to endothelial dysfunction and AS progression by generating ROS that damage endothelial cells and promote inflammation and lipid peroxidation (Halliwel et al., 2023). Ferroptosis, marked by iron-dependent lipid peroxidation, is linked to AS. It causes the death of endothelial cells and macrophages in atherosclerotic lesions, destabilizing plaques (Wang et al., 2021; Yang et al., 2022). Pro-inflammatory cytokines like TNF-α and IL-6 can worsen iron overload by upregulating HEP, which reduces FPN expression and increases iron retention in macrophages, further promoting oxidative stress (Izumi et al., 2023). Generally, the interplay between iron overload and ferroptosis exacerbates inflammation and plaque instability. Targeting these pathways with iron chelators or ferroptosis inhibitors may offer therapeutic strategies to mitigate AS progression and enhance cardiovascular health.

This study has several limitations. First, the small sample size may constrain statistical power and generalizability. Additionally, methodological constraints prevented a comprehensive analysis of cellular cross-talk, particularly the absence of *in vitro* experiments to dissect cell-type-specific mechanisms. Furthermore, we did not evaluate potential effects between TMP and PF, leaving the mechanisms of combination therapy unexplored. Moreover, the lack of mitochondrial electron microscopy experiments restricts our understanding of mitochondrial alterations associated with ferroptosis. Lastly, reliance on animal models inherently limits direct applicability to humans.

Our study adds to the current understanding of AS by suggesting an association between iron metabolism, ferroptosis-related markers, and disease progression. Our findings indicate that TMP and PF may modulate these pathways, offering preliminary insights into potential novel therapeutic strategies that may reduce inflammation and oxidative stress by addressing iron dysregulation and ferroptosis-associated processes. Furthermore, our results point to the possible value of integrating traditional botanical remedies such as TMP and PF with modern therapeutic practices in a holistic approach for AS management.

## 5 Conclusion

In conclusion, this study offers insights into the potential involvement of iron metabolism and ferroptosis-related pathways in the pathophysiology of AS. Our findings suggest that combination strategies incorporating both traditional and modern therapeutic approaches may offer promise for cardiovascular health, and may inform future development of natural metabolite-based interventions for AS. Further studies are warranted to validate the functional role of ferroptosis in these observed effects and to establish definitive therapeutic efficacy.

## Data Availability

The datasets presented in this study can be found in online repositories. The names of the repository/repositories and accession number(s) can be found in the article/Supplementary Material.
