# Supplementary material for: The role of tetramethylpyrazine and paeoniflorin in modulating iron metabolism and ferroptosis: innovative strategies for atherosclerosis treatment
Source: Front Pharmacol. 2026 Jul 13;17:1845893. doi: 10.3389/fphar.2026.1845893 (PMC13402380; doi:10.3389/fphar.2026.1845893)
Supplement: Supplementary file 1 [file Table1.docx]

**Table S1. Primer sequences for qRT-PCR**

| **Gene** | **Primer sequence** (5'→3') |
| --- | --- |
| GAPDH | F: GAAGGTGAAGGTCGGAGT R: GAAGATGGTGATGGGATTTC |
| Hepcidin | F: CCTGACCAGTGGCTCTGTTT  R: CACATCCCACACTTTGATCG |
| FPN | F: ACCTCGCTGGTGGTACAGAATGTT  R: AGCAGGAAGTGAGAACCCATCCAT |
| PTGS2 | F: TGGTCTGGTGCCTGGTCTGATG  R: CCTGCTTGTCTGGAACAACTGCTC |
| GPX4 | F: GAGGCAAGACCGAAGTAAACTAC  R: CCGAAACTGGTTACACGGGAA |
| P53 | F: TGTAGCTGAGACTCCTGCA  R: AACCTCAGGTGGCTCATAG |
| FTH1 | F: CCCCCATTTGTGTGACTTCAT  R: GCCCGAGGCTTAGCTTTCATT |
| SCL7A11 | F: GGCTCCATGAACGGTGGTGTG  R: GCTGGTAGAGGAGTGTGCTTGC |
| NOX1 | F: TTTGTCGGCCTTCTCATATT  R: GAATCTTCCCTGTTGCCTAGAA |
