# Supplementary material for: The role of tetramethylpyrazine and paeoniflorin in modulating iron metabolism and ferroptosis: innovative strategies for atherosclerosis treatment
Source: Front Pharmacol. 2026 Jul 13;17:1845893. doi: 10.3389/fphar.2026.1845893 (PMC13402380; doi:10.3389/fphar.2026.1845893)
Supplement: Supplementary file 3 [file Table2.docx]

**Table S2. Taxonomic authentication of plant species (with authorities and families)**

| **Species (with authorities & family)** | **Region** | **Pharmacopeial Drug Name** | **Plant Part** | **Key Markers** |
| --- | --- | --- | --- | --- |
| Ligusticum chuanxiong Hort. (syn. Conioselinum anthriscoides (H.Boissieu) Pimenov & Kljuykov; Ligusticum sinense Oliv.) **Family: Apiaceae** | China | Chuanxiong Rhizoma (川芎) | Rhizome | Ligustilide (≥0.3%), Ferulic acid (≥0.02%) |
|  | Japan | Senkyu (センキュウ) | Rhizome | Senkyunolide A (≥0.2%) |
|  | Europe | Ligusticum Root | Root & Rhizome | Volatile oil (≥1.5% v/w) |
| Paeonia lactiflora***Pall*. Family: Paeoniaceae** | China | Chishao (赤芍) | Unpeeled root | Paeoniflorin (≥1.8%) |
|  | Korea | Jakhak (작약) | Peeled root | Albiflorin (≥0.5%) |
