# Supplementary figures and images for: The role of tetramethylpyrazine and paeoniflorin in modulating iron metabolism and ferroptosis: innovative strategies for atherosclerosis treatment
Source: Front Pharmacol. 2026 Jul 13;17:1845893. doi: 10.3389/fphar.2026.1845893 (PMC13402380; doi:10.3389/fphar.2026.1845893)

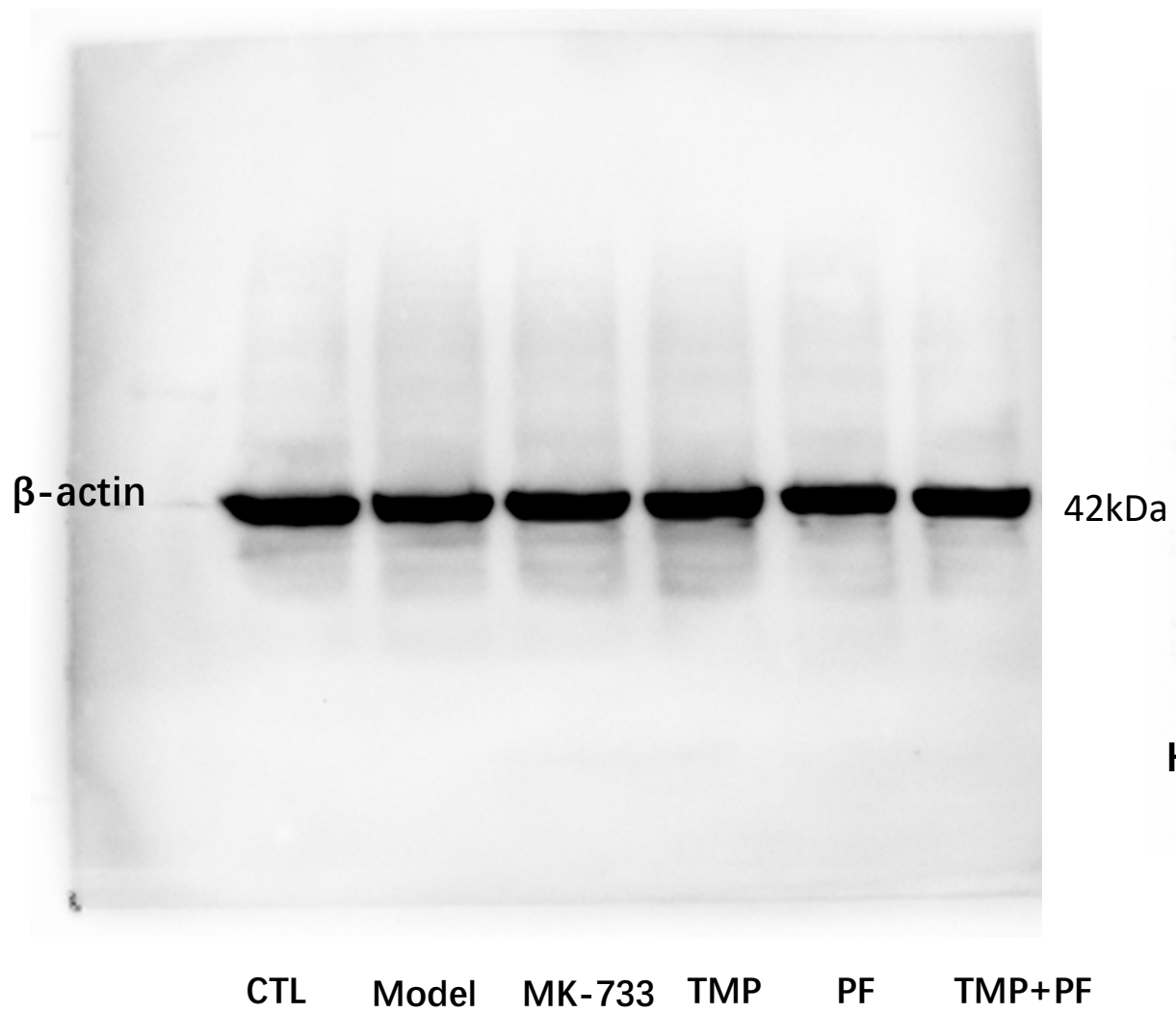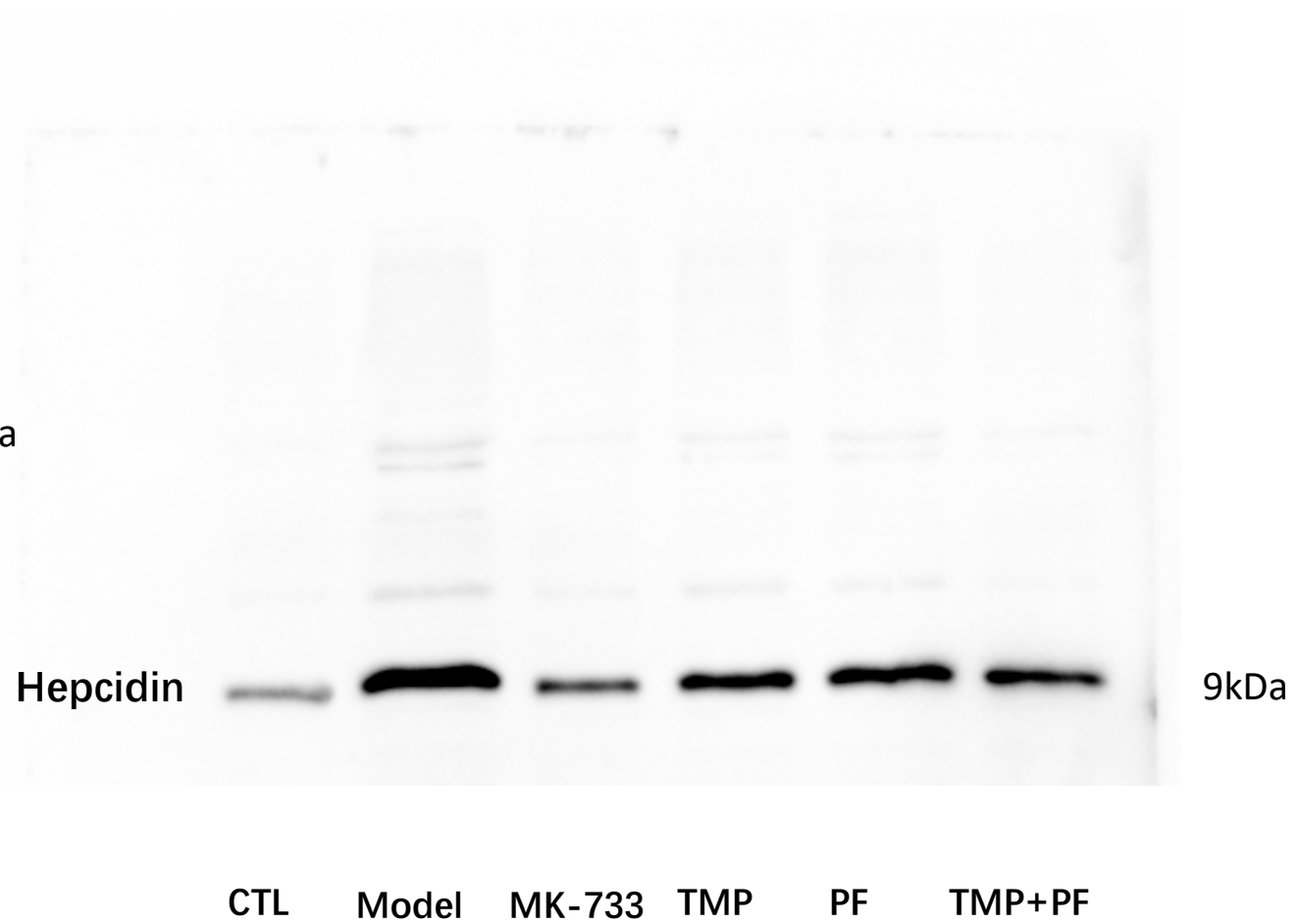

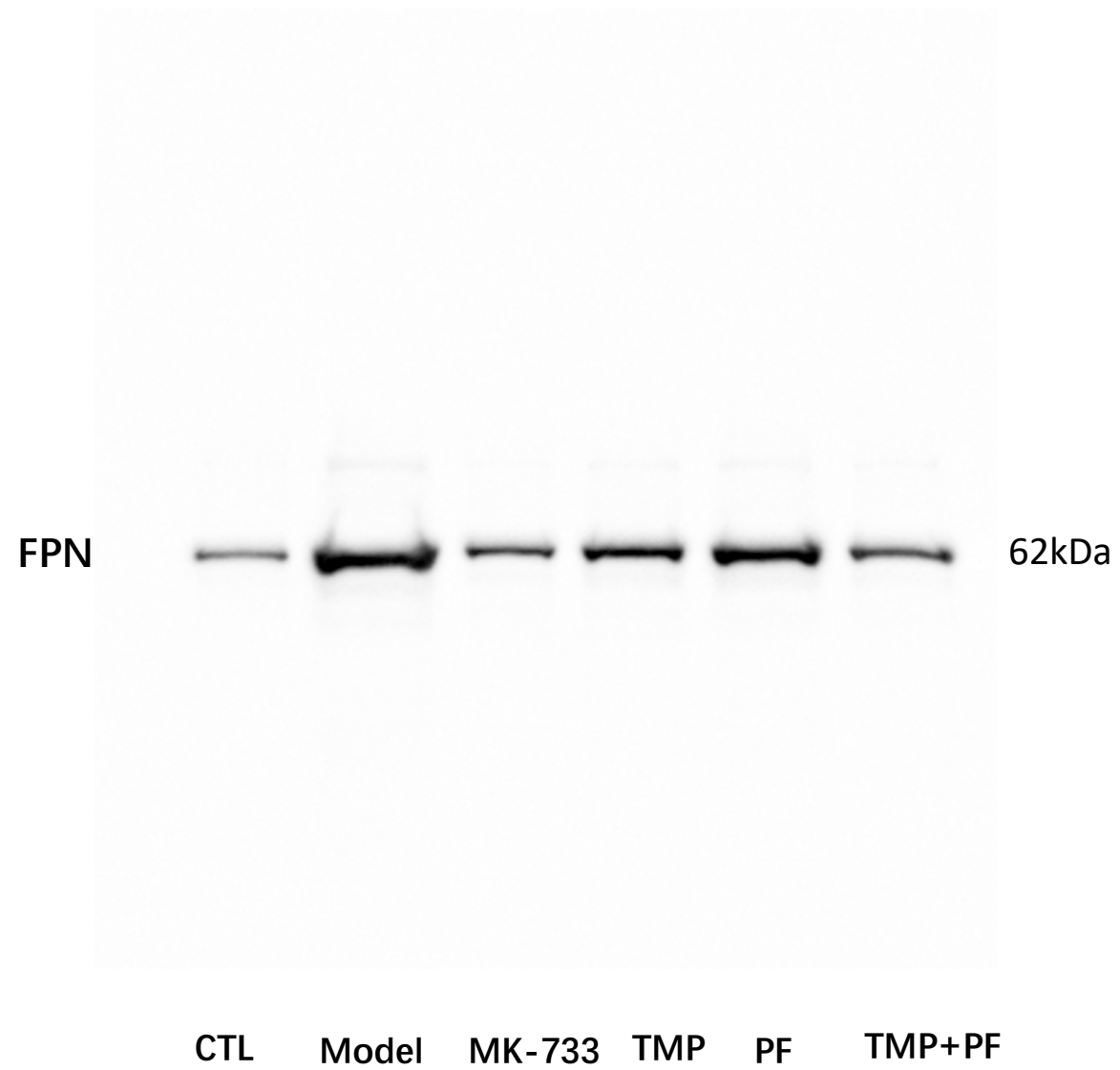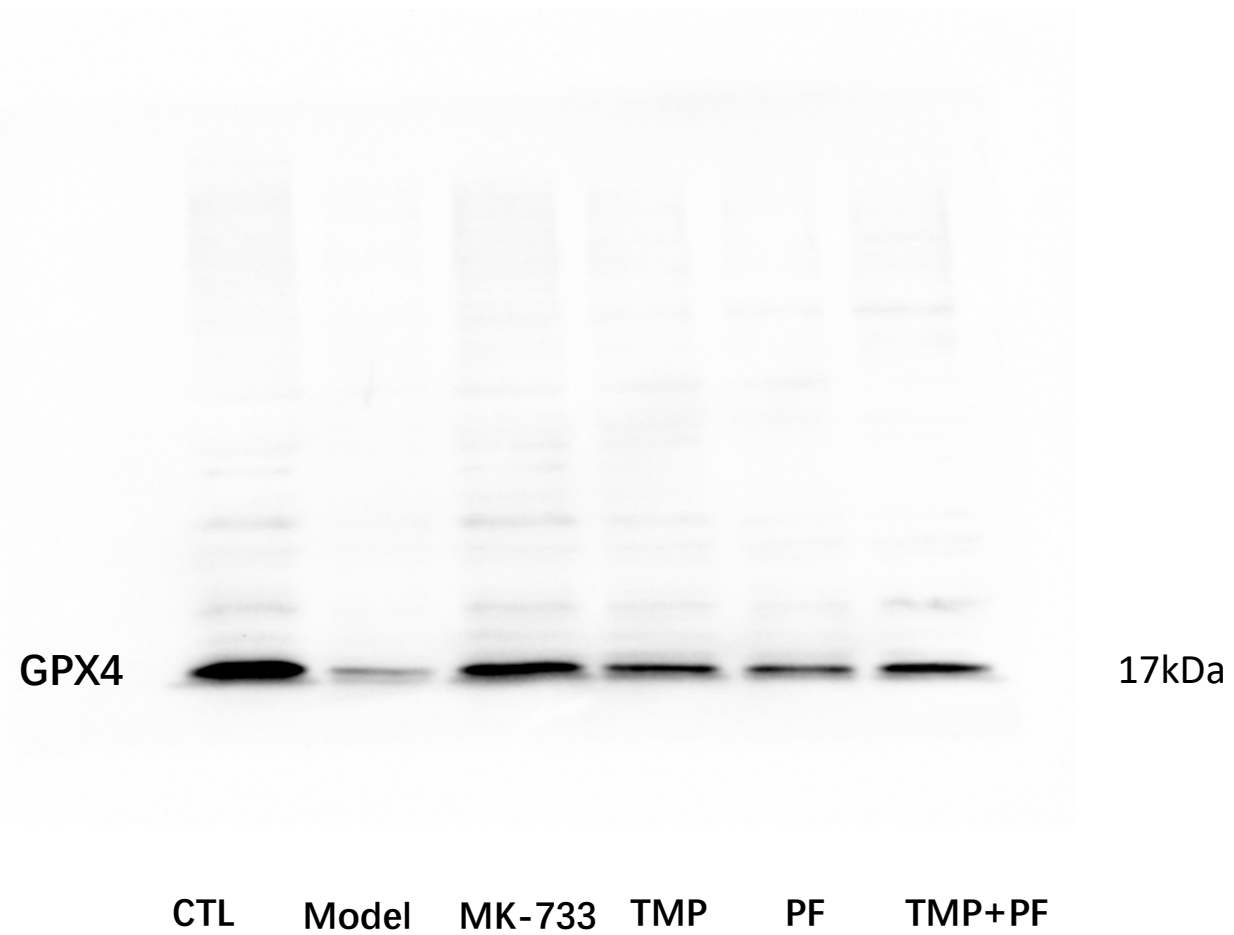

P53

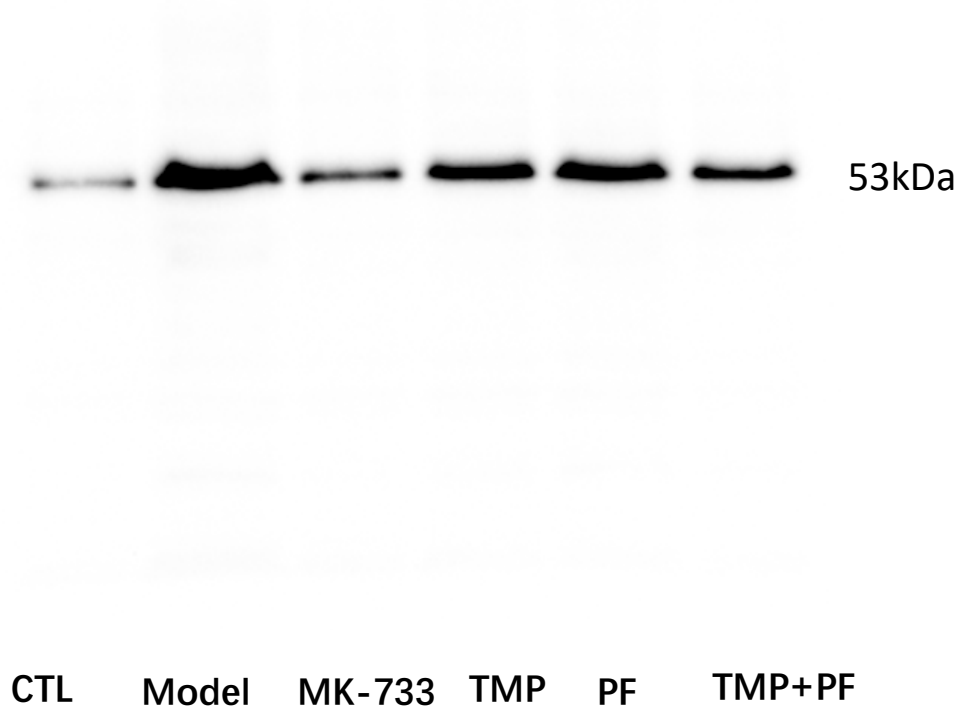

SLC7A11

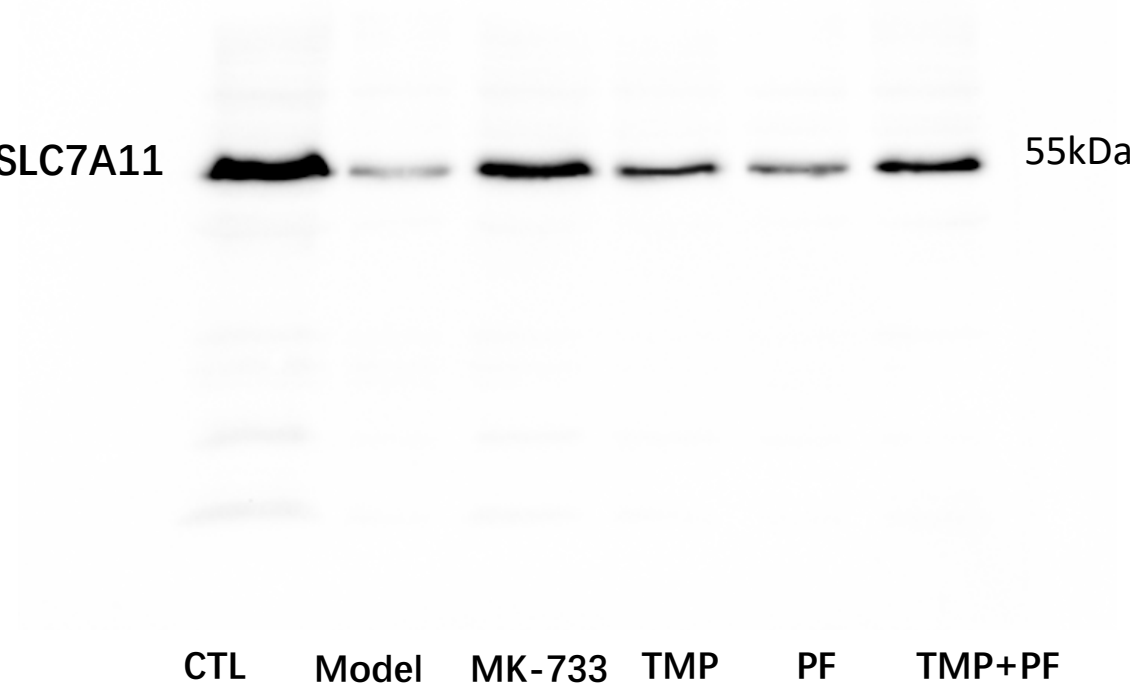

PTGS2 69kDa

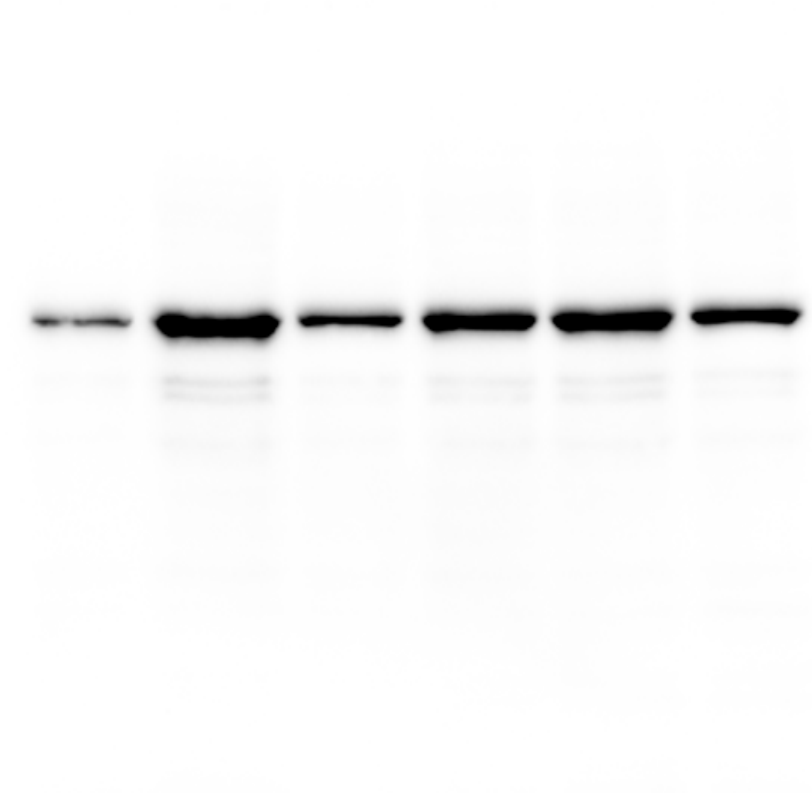

CTL    Model    MK-733    TMP    PF    TMP+PF

NOX1 65kDa

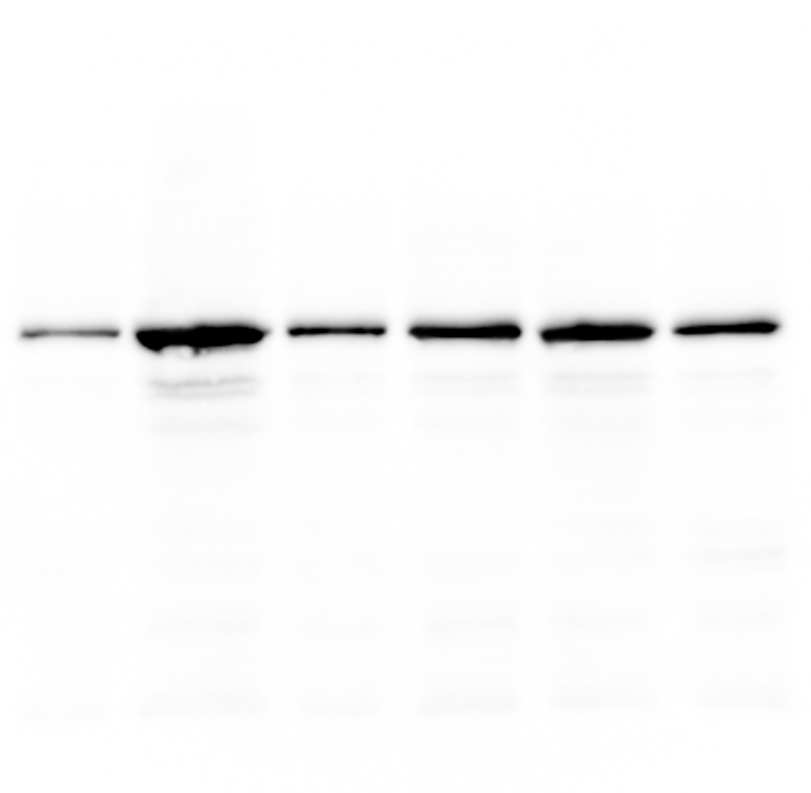

CTL    Model    MK-733    TMP    PF    TMP+PF

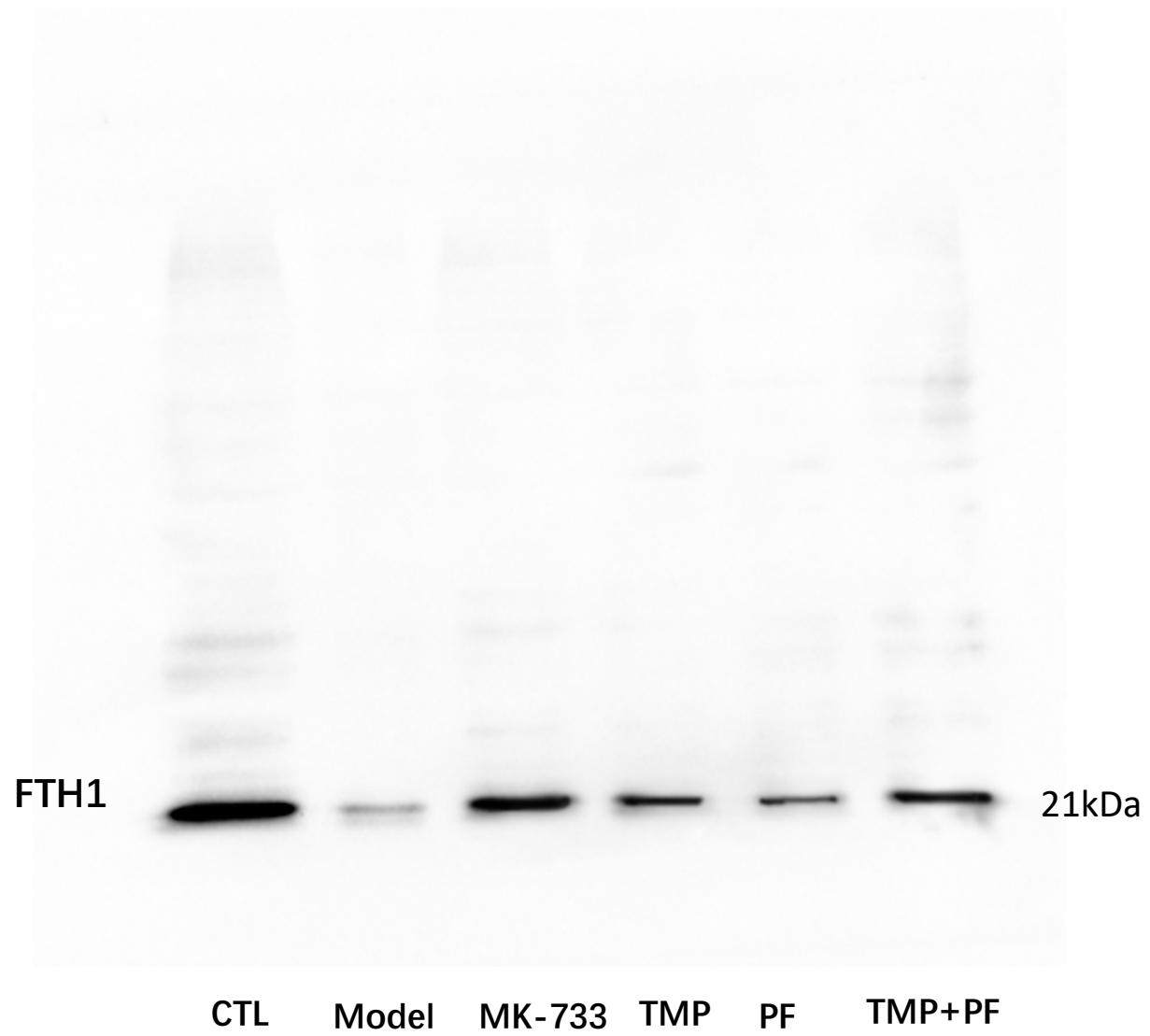

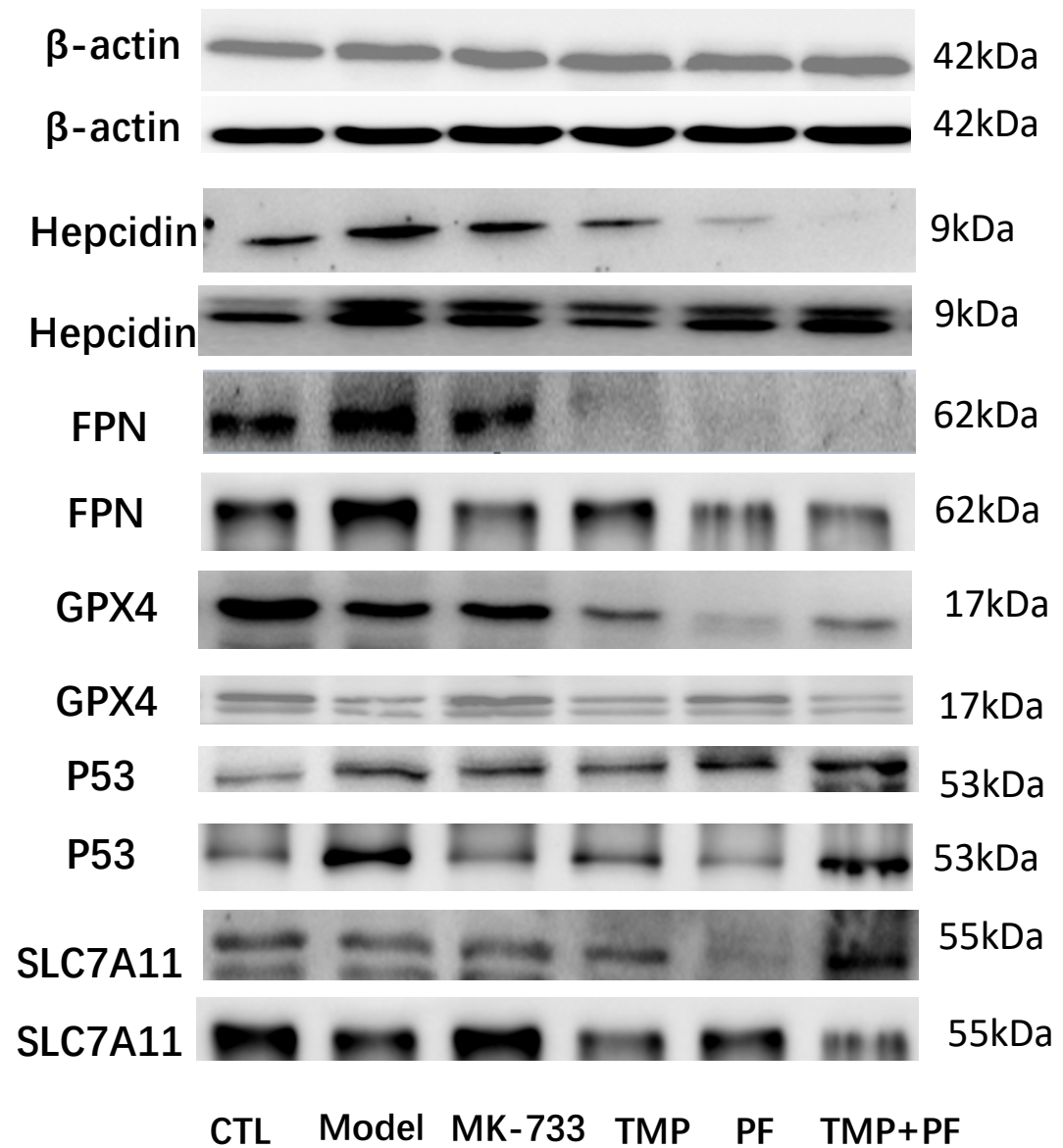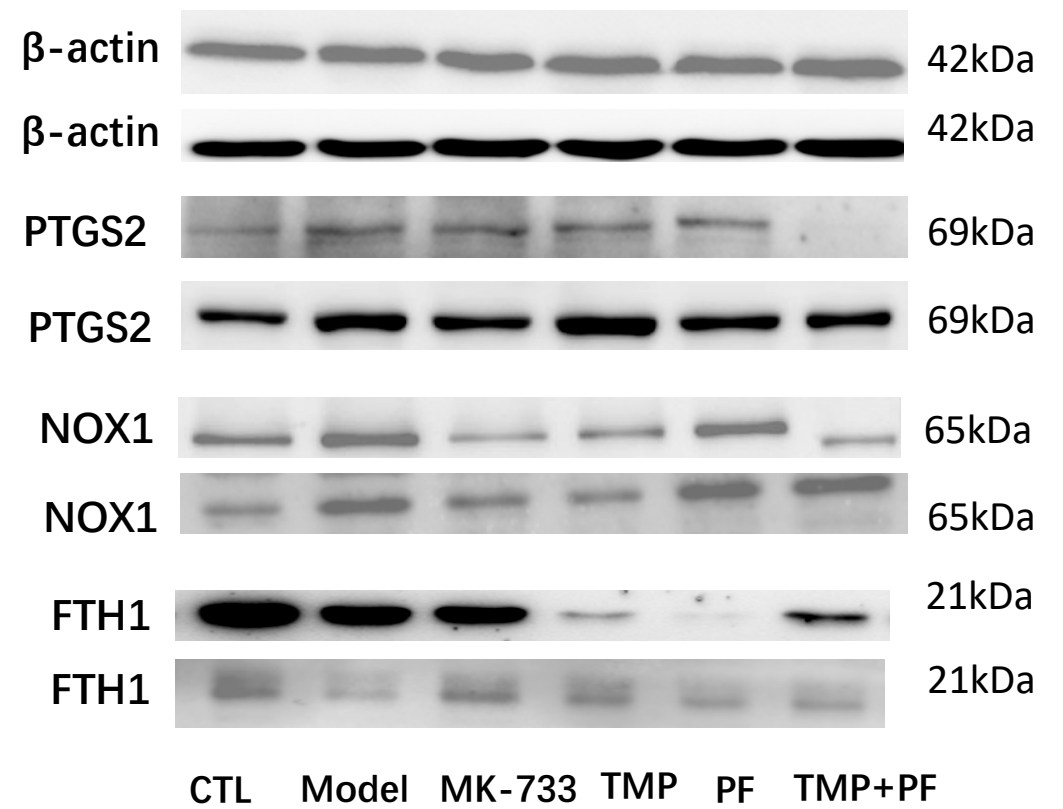

Supplement: Supplementary file 4 [file Supplementaryfile2.pdf]
